# Short-term efficacy and safety of nondamaging retinal laser therapy for retinitis pigmentosa-associated cystoid macular edema

**DOI:** 10.55730/1300-0144.6012

**Published:** 2025-04-17

**Authors:** Seda ÇEVİK KAYA, Mehmet ÇITIRIK, Mevlüt YILMAZ, Eyüpcan ŞENSOY

**Affiliations:** Department of Ophthalmology, University of Health Sciences, Ankara Etlik City Hospital, Ankara, Turkiye

**Keywords:** Retinitis pigmentosa, cystoid macular edema, nondamaging retinal laser therapy, central macular thickness, visual acuity, micropulse laser

## Abstract

**Background/aim:**

To evaluate the short-term efficacy and safety of nondamaging retinal laser therapy (NRT) for retinitis pigmentosa-associated cystoid macular edema (RP-CME) and analyze anatomical and functional outcomes.

**Materials and methods:**

A retrospective case series of 40 eyes from 30 patients with RP-CME was conducted. The patients underwent NRT using a PASCAL laser system with endpoint management (EpM) software. Central macular thickness (CMT) and best-corrected visual acuity (BCVA) were assessed at baseline and 2 months posttreatment. Long-term follow-up data, including recurrence rates were also reviewed.

**Results:**

At the 2-month follow-up, NRT significantly reduced mean CMT by 79.7 μm (p < 0.001) and improved BCVA by 0.10 LogMAR (p < 0.001). Complete resolution of CME was observed in 30% of the eyes, while 52.5% experienced recurrence within 6 months. Unilateral cases exhibited greater CMT reductions than bilateral cases (p < 0.05). No retinal damage from the laser was observed. The long-term sustainability of these effects remains unclear, and repeated treatments have not yet been assessed.

**Conclusion:**

NRT is a safe and effective short-term treatment for RP-CME, achieving significant anatomical and functional improvements without evidence of retinal damage. However, the high recurrence rate and absence of long-term data warrant further investigation. Future studies should explore repeated treatments, genetic subtypes, and correlations with the ellipsoid zone integrity.

## 1. Introduction

Retinitis pigmentosa (RP) is a prevalent hereditary retinal disorder characterized by the progressive degeneration of photoreceptors and subsequent loss of peripheral visual fields. While central vision is frequently preserved until advanced stages of the disease, up to 10%–50% of patients develop cystoid macular edema (CME), which can significantly impair central vision and quality of life. The underlying pathophysiology of RP-associated CME (RP-CME) remains unclear, but it is hypothesized to involve a combination of retinal pigment epithelium (RPE) dysfunction, Müller cell impairment, and blood-retinal barrier breakdown [1–5].

Early identification and management of CME in RP are crucial, since persistent edema may result in permanent retinal damage and further decline in visual acuity (VA) [6]. Current therapeutic strategies include carbonic anhydrase inhibitors (CAIs), intravitreal corticosteroids, antivascular endothelial growth factor (anti-VEGF) injections, and micropulse laser therapy [7–12]. Nonetheless, these interventions frequently have limited effectiveness, and many carry significant risks, such as glaucoma or endophthalmitis. Therefore, there is a growing demand for alternative, minimally invasive, and safe treatment options for RP-CME.

Nondamaging retinal laser therapy (NRT), a subthreshold photothermal method, has gained recognition as a potentially effective intervention for macular edema in other retinal disorders, such as diabetic macular edema (DME) and central serous chorioretinopathy (CSCR) [13–15]. Unlike traditional photocoagulation, NRT activates RPE cells and promotes heat shock protein (HSP) production without causing observable retinal injury. This process is believed to normalize fluid balance and guard against apoptosis, potentially alleviating CME without damaging the outer layers of the retina [16,17]. Although it has shown promising results in other contexts, the supporting evidence for NRT in RP-CME remains limited.

This study aimed to evaluate the short-term effects of NRT on central macular thickness (CMT) and best-corrected visual acuity (BCVA) in patients with RP-CME. By addressing this research gap, we aim to determine whether NRT offers a safe, effective, and cost-efficient alternative for managing this challenging condition.

## 2. Methods

This study was a retrospective interventional case series conducted at a tertiary referral center between January 2020 and September 2022. This study adhered to the tenets of the Declaration of Helsinki and was approved by the Local Research Ethics Committee (Approval ID: AEŞH-EK1-2023-078). Written informed consent was obtained from all participants prior to any invasive procedure.

Clinical records were reviewed for patients diagnosed with retinitis pigmentosa (RP) who developed cystoid macular edema (CME) during follow-up. Subjects were included if they met the following criteria: presence of intraretinal cysts confirmed by spectral-domain optical coherence tomography (SD-OCT), CME resistant to systemic or topical carbonic anhydrase inhibitors (CAIs), no intravitreal therapy in the preceding 3 months, and a minimum follow-up of 6 months posttreatment.

Exclusion criteria included the presence of subretinal fluid or other causes of CME (e.g., uveitis, epiretinal membrane, diabetic maculopathy, or retinal vein occlusion), any inflammatory or infectious eye conditions, a history of retinal laser photocoagulation or intraocular procedures (except uncomplicated cataract surgery), any inherited or acquired retinal disorder other than RP, and cataracts or other media opacities that compromised OCT image quality.

All patients underwent nondamaging retinal laser therapy (NRT) using a PASCAL pattern-scanning laser system (Optimedica Corp., Santa Barbara, CA, USA) with endpoint management (EpM) software. The treatment was performed by a single retinal specialist (M.C.) using a 577 nm yellow laser. The laser parameters included a spot size of 200 μm, a pulse duration of 15 ms, and a 0.5-spot spacing. The laser power was titrated starting at 100 mW until a barely visible burn was observed outside the vascular arcade. The treatment power was then set to 30% of the barely visible burn threshold. A Volk Area Centralis lens (Volk Optical Inc., Mentor, OH, USA) was used for all procedures.

The primary endpoints were central macular thickness (CMT) and best-corrected visual acuity (BCVA). CMT was measured in micrometers using manual segmentation on SD-OCT (Spectralis; Heidelberg Engineering, Heidelberg, Germany), extending from the internal limiting membrane to Bruch’s membrane. BCVA was assessed using a Snellen chart and converted into logarithm of the minimum angle of resolution (LogMAR) notation.

Secondary endpoints included the presence of CME (categorized as complete resolution, persistence, or recurrence) and safety evaluation through fundus examination and OCT. Recurrence was defined as an increase in CMT ≥ 20 μm from the lowest posttretment value.

Patients were evaluated at baseline and at 2 and 6 months posttreatment. For cases followed beyond 6 months, additional data on CME recurrence and progression were collected.

Data analysis was performed using SPSS software (version 23.0; IBM, Armonk, NY, USA). Descriptive statistics are presented as means ± standard deviations (SD). Normality of data was assessed using the Kolmogorov–Smirnov test, which showed normal distribution (p > 0.05). Changes in BCVA and CMT were analyzed using the Wilcoxon signed-rank test. Differences between unilateral and bilateral cases were evaluated using the Mann–Whitney U test. Relationships between variables were analyzed using Spearman’s rank correlation coefficients. Categorical data were analyzed using Pearson’s chi-squared test. Statistical significance was set at p < 0.05.

## 3. Results

The study included 40 eyes from 30 patients, with 20 patients having unilateral involvement and 10 with bilateral CME. The mean age of the patients was 35.82 ± 6.58 years (range: 22–48 years), with an equal male-to-female ratio (1:1). The average duration from onset of RP-associated CME to NRT was 13.1 ± 6.5 months.

At baseline, the mean central macular thickness (CMT) was 311.80 ± 54.94 μm, and the mean best-corrected visual acuity (BCVA) was 0.43 ± 0.13 LogMAR. At the 2-month follow-up, CMT was significantly reduced to 232.10 ± 50.60 μm (mean reduction: 79.7 μm; p < 0.001) and BCVA improved to 0.33 ± 0.19 LogMAR (p < 0.001). The mean CMT and BCVA values at baseline and after nondamaging retinal laser therapy are presented in Table. Complete resolution of CME was observed in 12 eyes (30%), while 28 eyes (70%) exhibited partial persistence.

Unilateral cases showed a greater reduction in CMT (mean: 89.2 μm) than bilateral cases (mean: 70.2 μm; p < 0.05). However, there was no statistically significant difference in BCVA improvement between unilateral and bilateral cases (p = 0.43).

CME recurrence occurred in 21 eyes (52.5%) within 6 months. Most recurrences were in eyes with partial initial response, though some were also noted in eyes with complete resolution. Recurrence was defined as an increase in CMT ≥ 20 μm from the lowest posttreatment value.

No significant correlations were found between CME status at 2 months and patient age (p = 0.63), baseline CMT (p = 0.27), or symptom duration (p = 0.64). Mean follow-up duration after NRT was 9.05 ± 1.06 months.

Importantly, no laser-induced retinal damage, such as scarring or photoreceptor disruption, was observed on fundus examination or OCT in any patient.

**Figure 1:**
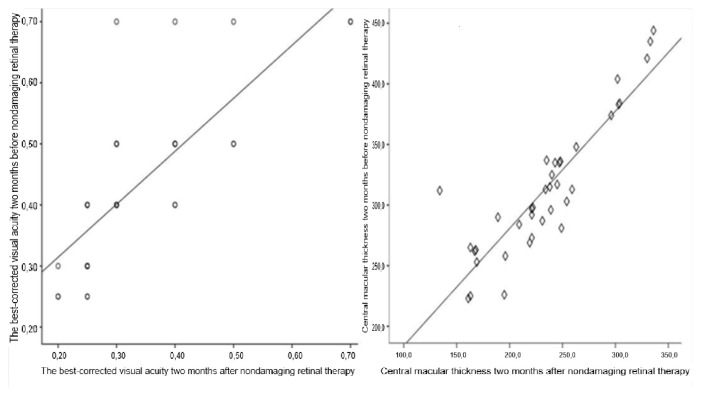
Association between baseline and posttreatment BCVA and CMT. Scatter plots showing the relationship between baseline and 2-month posttreatment central macular thickness (CMT) (left) and best-corrected visual acuity (BCVA) (right) after nondamaging retinal laser therapy (NRT). The data demonstrate that outcomes are influenced by baseline anatomical and functional status.

**Figure 2:**
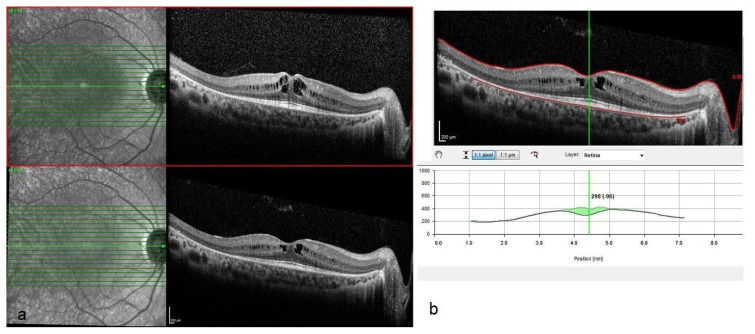
OCT imaging of a case with significant CMT reduction after NRT. (Example 1). Spectral-domain optical coherence tomography (SD-OCT) images of a representative patient before and 2 months after NRT. (a) Baseline OCT showing significant cystoid macular edema with CMT of 456 μm. (b) Posttreatment OCT showing substantial resolution of cystoid spaces and a CMT reduction of 298 μm.

**Figure 3:**
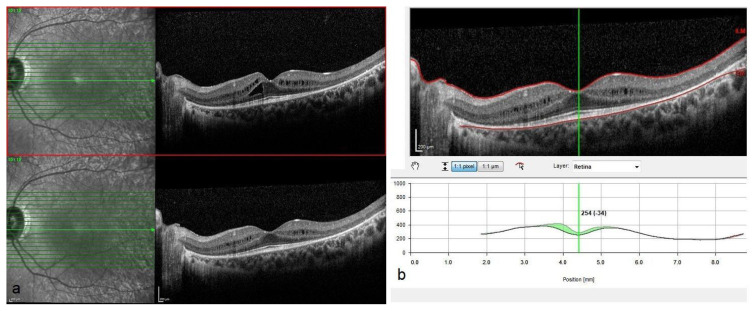
OCT imaging of a case with moderate CMT reduction after NRT. (Example 2). (a) Baseline OCT showing CME with a central macular thickness of 418 μm. (b) Posttreatment OCT demonstrating partial resolution of cystoid spaces, with a 254 μm reduction in CMT.

Representative optical coherence tomography (OCT) images and associated changes in central macular thickness (CMT) before and after NRT treatment are shown in Figures 1–3. Figure 1 demonstrates the relationship between baseline and posttreatment best-corrected visual acuity (BCVA) and CMT, highlighting the influence of initial anatomical and functional status on clinical outcomes. Figure 2 illustrates a case with substantial anatomical improvement: Figure 2a shows pronounced cystoid macular edema with a baseline CMT of 456 μm, while Figure 2b reveals a marked resolution of the cystic spaces and a 298 μm reduction in CMT 2 months after NRT. A similar response is depicted in Figure 3, where baseline OCT in Figure 3a shows a CMT of 418 μm, and Figure 3b reveals significant regression of cystoid changes, corresponding to a 254 μm decrease in CMT posttreatment.

## 4. Discussion

Nondamaging retinal laser therapy (NRT) aims to deliver subthreshold thermal stimulation to the retina without causing permanent tissue injury, thereby activating protective and reparative cellular pathways. Specifically, the mild hyperthermia induces upregulation of heat shock proteins (HSPs), which assist in refolding misfolded proteins and suppress apoptotic signaling. In the context of retinitis pigmentosa (RP), this sublethal stress may help restore or enhance retinal pigment epithelium (RPE) functions—such as fluid transport—contributing to the reduction of cystoid edema. Additionally, NRT has been shown to stimulate the expression of glial fibrillary acidic protein (GFAP) in Müller cells, suggesting a remodeling response that could stabilize photoreceptors. Unlike conventional photocoagulation, which causes visible retinal damage, NRT produces no detectable lesions and can be safely repeated, making it a promising long-term strategy for managing RP-related cystoid macular edema (CME) and other macular disorders [15,16].

This study demonstrated that NRT is a safe and potentially beneficial short-term treatment for RP-related CME, with significant reductions in central macular thickness (CMT) and improvements in best-corrected visual acuity (BCVA) observed at 2 months posttreatment. Notably, 30% of eyes showed complete CME resolution without signs of retinal damage. These findings are consistent with results from previous NRT applications in diabetic macular edema (DME) and central serous chorioretinopathy (CSCR), supporting the hypothesis that subthreshold laser can modulate RPE function and retinal fluid dynamics without structural injury [18–20].

When compared to other therapeutic options—including carbonic anhydrase inhibitors (CAIs), intravitreal corticosteroids, anti-VEGF agents, and micropulse laser—NRT offers a favorable safety and tolerability profile. While CAIs may lead to anatomical improvement, their effect on visual function is often limited. Steroids and anti-VEGF injections involve procedural risks such as endophthalmitis and elevated intraocular pressure, and their efficacy can diminish over time [21]. Micropulse laser, though safer than traditional photocoagulation, still carries a risk of delivering higher thermal energy than NRT. The noninvasive and repeatable nature of NRT positions it as an attractive adjunct or alternative strategy in the long-term management of RP-CME.

A key challenge observed in our study is the high recurrence rate of 52.5% within 6 months. This rate is comparable to those reported for CAIs and corticosteroid regimens in RP-CME, where recurrence often occurs within months of treatment discontinuation. Data on anti-VEGF recurrence in RP-related CME are limited and variable, but also suggest that treatment effects may be temporary [22–24]. Recurrence was more frequent in bilateral CME cases, potentially reflecting greater disease severity or systemic influences. These findings emphasize the need for more durable therapeutic strategies and for future studies evaluating repeated NRT applications, as have been safely conducted in CSCR.

The pathogenesis and responsiveness of RP-CME are influenced by a range of factors that remain incompletely understood. Although age, sex, and pseudophakia have been studied, their associations with CME risk in RP patients remain inconclusive. Genetic heterogeneity likely plays a significant role. CME has been reported across all RP subtypes, with prevalence and severity varying based on genotype. Stratifying patients by genetic mutation class or anatomical markers such as ellipsoid zone integrity may help predict responsiveness to NRT and guide personalized treatment.

Designing randomized controlled trials (RCTs) to assess NRT efficacy presents ethical and logistical challenges. Sham laser arms, while ideal methodologically, are difficult to implement in visually impaired populations. Simulating treatment without therapeutic intent raises ethical concerns, and patient masking may be unreliable. These issues complicate the generation of high-level evidence, but do not diminish the need for rigorous, prospective studies.

Beyond these structural and genetic considerations, several external variables may further influence outcomes, including concurrent use of dietary supplements or off-label treatments and differences in adherence or follow-up compliance. These factors highlight the need for large, multicenter RCTs with standardized protocols, longer follow-up, and stratification by genotype. Such studies are critical for confirming NRT’s effectiveness and integrating it into broader treatment frameworks.

Despite the limitations of this study—including its retrospective nature, absence of a control group, small sample size, relatively short follow-up, and lack of fluorescein angiography—the current findings provide encouraging evidence supporting the short-term safety and efficacy of NRT in RP-related CME. These results suggest that NRT may be a valuable adjunct in selected patients, particularly those with early-stage or unilateral disease. Further validation in prospective trials is necessary to define its long-term role.

In conclusion, nondamaging retinal laser therapy appears to be a safe and effective option for treating CME associated with RP. It provides short-term anatomical and functional improvements without inducing retinal damage. However, recurrence remains a concern and long-term outcomes are unclear. Future studies should explore repeated treatments, patient stratification by genetic and anatomical features, and strategies to sustain therapeutic benefits over time. Until stronger evidence is available, NRT should be considered a supplemental therapy for appropriately selected RP-CME patients.

## Figures and Tables

**Table:** Central foveal thickness and best-corrected visual acuity before and after NRT.

Measurement	Before NRT (mean ± SD)	After NRT (mean ± SD)	p-value
Central foveal thickness (μm)	311.80 ± 54.94	232.10 ± 50.60	p < 0.001
BCVA (LogMAR)	0.43 ± 0.13	0.33 ± 0.19	p < 0.001

BCVA: best-corrected visual acuity; LogMAR: logarithm of the minimum angle of resolution; NRT: nondamaging retinal laser therapy.
